# Transcriptional regulation of male-sterility in *7B-1* male-sterile tomato mutant

**DOI:** 10.1371/journal.pone.0170715

**Published:** 2017-02-08

**Authors:** Vahid Omidvar, Irina Mohorianu, Tamas Dalmay, Yi Zheng, Zhangjun Fei, Anna Pucci, Andrea Mazzucato, Vendula Večeřová, Michaela Sedlářova, Martin Fellner

**Affiliations:** 1 Laboratory of Growth Regulators, Centre of the Region Haná for Biotechnological and Agricultural Research, Palacký University and Institute of Experimental Botany AS CR, Šlechtitelů 27, Olomouc-Holice, Czech Republic; 2 School of Computing Sciences, University of East Anglia, Norwich, United Kingdom; 3 School of Biological Sciences, University of East Anglia, Norwich, United Kingdom; 4 Boyce Thompson Institute, Cornell University, Ithaca, NY, United States of America; 5 Department of Agricultural and Forestry Sciences, University of Tuscia, Viterbo, Italy; 6 Department of Botany, Faculty of Science, Palacký University in Olomouc, Šlechtitelů 27, Olomouc-Holice, Czech Republic; Wuhan University, CHINA

## Abstract

The *7B-1* tomato (*Solanum lycopersicum* L. cv Rutgers) is a male-sterile mutant with enhanced tolerance to abiotic stress, which makes it a potential candidate for hybrid seed breeding and stress engineering. To underline the molecular mechanism regulating the male-sterility in *7B-1*, transcriptomic profiles of the *7B-1* male-sterile and wild type (WT) anthers were studied using mRNA sequencing (RNA-Seq). In total, 768 differentially expressed genes (DEGs) were identified, including 132 up-regulated and 636 down-regulated transcripts. Gene ontology (GO) enrichment analysis of DEGs suggested a general impact of the *7B-1* mutation on metabolic processes, such as proteolysis and carbohydrate catabolic process. Sixteen candidates with key roles in regulation of anther development were subjected to further analysis using qRT-PCR and *in situ* hybridization. Cytological studies showed several defects associated with anther development in the *7B-1* mutant, including unsynchronized anther maturation, dysfunctional meiosis, arrested microspores, defect in callose degradation and abnormal tapetum development. TUNEL assay showed a defect in programmed cell death (PCD) of tapetal cells in *7B-1* anthers. The present study provides insights into the transcriptome of the *7B-1* mutant. We identified several genes with altered expression level in *7B-1* (including *beta-1*,*3 glucanase*, *GA2oxs*, *cystatin*, *cysteine protease*, *pectinesterase*, *TA29*, and *actin*) that could potentially regulate anther developmental processes, such as meiosis, tapetum development, and cell-wall formation/degradation.

## Introduction

In flowering plants, male-fertility is a highly regulated process, which requires proper cellular differentiation in anthers and timely regulation of microsporogenesis. Male-sterility on the other hand has potential application in hybrid seed breeding and understanding its molecular mechanism is currently an important research topic in plant science [1]. Large number of male-sterile tomato mutants have been identified, however, in most cases the mutant gene(s) have not been precisely identified and often mapped only to a large genomic region [1,2,3]. A *polygalacturonase* gene is the only well characterized gene known so far, which is responsible for male-sterile phenotype of *ps-2* tomato mutant [1]. Male-sterile tomato mutants with desired agricultural traits are advantageous for hybrid seed breeding. Male-sterile mutants in tomato have been classified into functional, structural, and sporogenous classes [4]. For example, *positional sterile-2* (*ps-2*) tomato is a functional male-sterile mutant with defected pollen dehiscence [1]. *Stamenless-2* (*sl-2*) tomato is a structural mutant, which produces abnormal stamens with aborted microspores [5]. In sporogenous mutants, microsporogenesis could break down during meiosis, formation of tetrads or separation of microspores. In *male-sterile* (*ms*) *3* and *ms15* tomato mutants, pollen mother cells (PMC) collapse in pre-meiotic anthers [6]. In *ms5* and *ms1035* (allelic to *ms10*) tomato mutants, microsporogenesis beaks down at meiosis due to aberrant regulation of tapetal cells [4,7].

Several genes with key roles in anther development have been characterized in *Arabidopsis*, among those, *SPL*/*NZZ*, *EMS1*/*EXS*, and *TPD1* are essential for differentiation of anther wall cells [8–11], and *MS1* and *MS2* are required for pollen wall formation [11,12]. In rice, *GAMYB* [13], *MYB33*/*MYB65* [14], *DYT1* [15], *TDF1* [16], *AMS* [17,18], *MS1* [19], *PTC1* [20], *TDR-2* [21], *UDT1* [22], *TDR* [23], and *EAT1* [24] play key roles in tapetum development and regulation of microsporogenesis. Studies in tomato and rapeseed suggest that male-sterility is, in part, a manifestation of hormonal imbalance in flowers, particularly in stamens [25–27]. Male-sterility is also known to be regulated by environmental factors, i.e., temperature, and photoperiod [28,29], and it has been suggested that the effects of these external agents are mediated through hormonal changes [26].

In most angiosperms, the anther consists of four lobes, each containing four highly specialized layers (from outer to inner: epidermis, endothecium, middle layer and tapetum), which houses the reproductive cells [30]. The tapetal cells play an important physiological role as all nutritional materials entering the sporogenous cells either passes through or originates from the tapetum [31]. In addition, the tapetum produces callase, an enzyme which removes the callose around tetrads. Aberrant regulation of tapetum development has been often associated with male-sterile anther phenotypes [32]. Tapetum degeneration is proposed to be triggered by PCD processes during the late stage of pollen development, which in turn provide cellular contents supporting pollen wall formation and maturation. Rice *TDR* mutant exhibits delayed tapetal PCD and retarded degeneration, resulting in male-sterility [32].

The *7B-1* tomato mutant line (*Solanum lycopersicum* L. cv. Rutgers) was previously described as a photoperiod-dependent male-sterile line [33,34]. In long days (LD), the *7B-1* flowers are male-sterile, which produce shrunken stamens with no viable microspores, while in short days (SD), flowers are fertile, stamens are intact and produce viable pollen. Compared to the WT, the mutant shows reduced de-etiolation, has higher content of endogenous Abscisic acid (ABA), but less gibberellins (GAs), indole-3-acetic acid (IAA), and cytokinins (CKs), and is hypersensitive to exogenous ABA [35–37]. Seed germination and hypocotyl growth in *7B-1* mutant are more tolerant to various abiotic stresses, especially under blue light [36]. Molecular studies showed defects in blue light perception and hormonal balance in the *7B-1* mutant, associated with a large number of proteins being differentially expressed between *7B-1* and WT anthers [36,38]. A recent study by Omidvar and Fellner [39] showed distinct DNA methylation dynamics and transcriptional regulation in response to different light qualities and abiotic stresses between *7B-1* and WT seedlings. Several microRNAs (miRNAs) with key roles in regulation of anther development, male-sterility and stress-response in *7B-1* have been identified and characterized [40,41]. With primary effect of the *7B-1* mutation yet unknown, studies indicate that modulation of the *7B-1* mutation and its effect on the gene expression is coordinated through a complex interplay between light signalling components, hormonal balance and their crosstalk with miRNAs and DNA methylation programming, which all collectively tune the downstream gene expression associated with anther development and male-sterility in *7B-1* anthers.

The aim of our study is to gain a deeper insight into the molecular mechanism of male-sterility and transcriptional regulation of anther developmental processes in *7B-1* anthers. Using RNA-Seq, we identified a number of genes with potential key roles in regulation of anther development and microsporogenesis, which were differentially expressed between WT and *7B-1* anthers. Expression profiles of these candidate genes were further investigated at different developmental stages of *7B-1* anthers using qRT-PCR and *in situ* hybridization. Cytological studies showed differences between WT and *7B-1* anthers, including anther structure, callose deposition and tapetum development.

## Materials and methods

### Plant materials

*7B-1* mutant and WT seedlings (*Solanum lycopersicum* L., cv. Rutgers) were grown in long days (16/8 h light/dark) in temperature controlled growth chamber. Flower buds at different developmental stages, including buds smaller than 4–5 mm (pre-meiotic anthers; referred to as S1), equal to 4–5 mm (meiotic anthers; S2) and bigger than 4–5 mm (post-meiotic anthers; S3) were collected and anthers were dissected under a stereomicroscope. Stages of flower buds were selected according to Sheoran et al. [38] and confirmed by analysis of anther squashes. Gibberelic acid treatment was carried out by spraying (0.1 mM GA3) directly onto the *7B-1* buds at the panicle primordium stage and repeated once a week until the buds reached the length of ≥ 5 mm.

### RNA-seq analysis

Total RNA was extracted from WT and *7B-1* anthers at different stages using the RNeasy Plant Mini Kit (Qiagen). Samples were pooled separately in equimolar ratio and used for construction of sequencing libraries using the Truseq™ RNA Sample Prep Kit (Illumina, San Diego, CA, USA). Sequencing was carried out on the Illumina HiSeq™ 2000 platform. Short reads and low quality bases were trimmed using Trimmomatic [42]. The remaining reads were mapped to the ribosome RNA database [43] using bowtie [44], allowing up to 3 mismatches and rRNA-mapping reads were subsequently filtered out. The cleaned reads were then mapped (allowing 2 mismatches) to the tomato reference genome ITAG v2.5 release using TopHat2 [45]. Read counts were normalized using the FPKM (fragments per kilobase per million) approach [46]. Differential expression analysis was carried out using NOISeq [47] and presented as offset fold change (OFC), with an offset of 20 as described by Mohorianu et al. [48]. Genes with log_2_ (OFC) ≥ 1.5 and probabilities > 0.95 were identified as DEGs. Gene ontologies were assigned using the Blast2go tool (http://www.blast2go.com/b2ghome). Enrichment analysis was carried out using PANTHER [49].

### Quantitative PCR

qRT-PCR experiments were carried out using the SensiFAST SYBR Lo-ROX kit (Bioline). First-strand cDNAs were synthesized using the PrimeScript First Strand cDNA Synthesis kit (Takara). Gene-specific primers are listed in S2 Table. Data normalization was carried out using the *CAC* and *α-tubulin* housekeeping genes (data were shown only for *CAC*). PCR thermal cycles were set for initial denaturation at 95°C for 2 min, 40 cycles of 95°C for 5 s, followed by annealing/extension at 60°C for 20 s. Differential expression values were calculated as normalized fold changes of expression using the ΔΔCT method [50].

### Light microscopy

Cryosections were prepared as described previously [40]. In brief, flower buds were embedded in Paraplast^®^ Plus^TM^ and transversal sections of 8 μM thickness were cut using a Leica Ultracut R ultramicrotome (Leica Bensheim, Germany). Callose was detected by staining the tissue sections with 0.05% (w/v) aniline blue and visualized with fluorescence microscopy (λ_exc_ = 330-385nm, λ_em_ = 480nm; Olympus BX60). *In situ* hybridization assay was carried out as previously described [40]. Oligo-probes (S3 Table) with sequences complementary to the candidate genes and murine miR122a (as a negative control) were synthesized and DIG-labelled at 5'-end by Eastport (Eastport, Czech Republic). Probe concentration and hybridization temperature were experimentally optimized to 10 nM and 50°C, respectively. *In situ* localization signals were detected using light microscopy in a colorimetric-based reaction using DIG-specific antibodies coupled to alkaline phosphatase.

### TUNEL assay

Anther sections were washed in PBS (160 mM NaCl, 2.7 mM KCl, 8 mM Na_2_HPO_4_, 1.5 mM KH_2_PO_4_) for 5 min and incubated in 20 mg/mL proteinase K in proteinase K buffer (100 mM Tris-HCl, pH 8.0, and 50 mM EDTA) for 20 min at 37°C in a humid chamber. Sections were washed in PBS for 5 min and fixed in 4% (w/v) paraformaldehyde in PBS for 10 min. PBS wash was repeated twice, each for 5 min. Detection of nuclear DNA fragmentation was performed using Terminal deoxynucleotidyl transferase (TdT) dUTP Nick-End Labeling (TUNEL) assay (DeadEnd Fluorometric TUNEL system, Promega) according to the manufacturer’s instructions. Fluorescence signal in samples was analyzed by fluorescence microscopy (wavelength of 520 ± 20nm; Olympus BX60).

### Experimental design and statistical analysis

Experiments were conducted in three biological replicates and arranged in a completely randomized design. Analysis of the variance (ANOVA) and mean comparison using duncan new multiple range test (DNMRT p = 0.05) were carried out using the SAS software version 9.2.

## Results

### Callose degradation is perturbed in *7B-1* anthers

**Fig 1:**
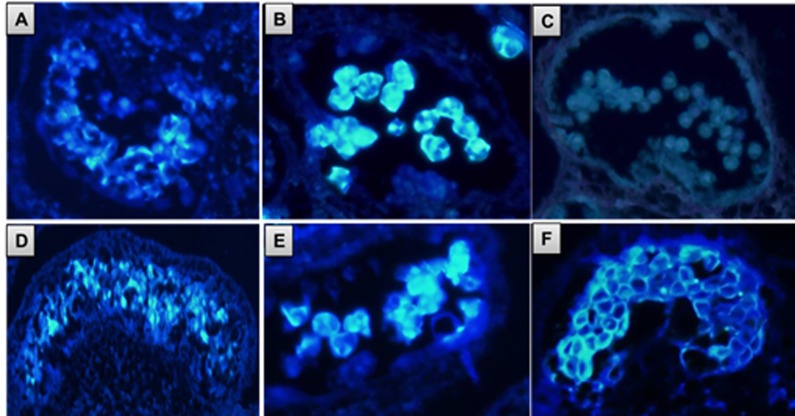
Callose deposition in WT (A, B, C) and *7B-1* (D, E, F) anthers. A, D: PMCs at early stage of meiosis. B, E: tetrad stage. C, F: microspores release stage.

We have previously showed that anther maturation in *7B-1* was not synchronized and microsporogenesis was impaired partially in some anthers/lobes as evidenced by arrested microspores. In addition, some anthers had abnormal tapetum phenotype, where the tapetal cells were vacuolated and failed to degenerate [41]. In this study, callose localization was examined in WT and *7B-1* anthers during meiosis (Fig 1). At the early PMC stage, callose was detected around the PMCs in both WT and *7B-1* anthers (Fig 1A and 1D). Callose was also detected in WT and *7B-1* meiotic anthers around the tetrads (Fig 1B and 1E). With release of microspores from the tetrads in WT anthers, callose was completely degraded as evidenced by lack of the signal, while it persisted around the arrested microspores in *7B-1* anthers (Fig 1C and 1F). This result showed that callose degradation was perturbed in *7B-1* anthers at the end of meiosis, resulting in the arrested microspore phenotype.

### Aberrant regulation of tapetum PCD in *7B-1* anthers

**Fig 2:**
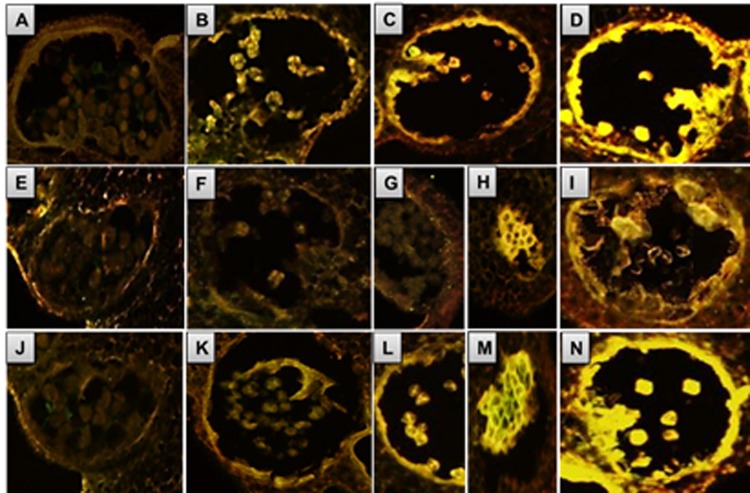
TUNEL assay in WT and *7B-1* anthers. Panels A, B, C, D: WT anthers at PMCs, tetrads, free binucleate microspores, and mature pollens stages, respectively. Panels E, F, G, H, I: *7B-1* anthers at PMCs, tetrads, free binucleate microspores, arrested binucleate microspores and mature pollens stages, respectively. Panels J, K, L, M, N: GA-treated *7B-1* anthers at the same stages as E-I.

The PCD in tapetal cells is characterized by cleavage of the nuclear DNA. To test if *7B-1* anthers are defective in PCD, we performed the TUNEL (terminal deoxynucleotidyl transferase–mediated dUTP nick-end labeling) assay (Fig 2). The assay measures nuclear DNA fragmentation, which can be visualized directly by fluorescence microscopy. Both WT and *7B-1* anthers undergoing meiosis showed TUNEL-negative signal (Fig 2A and 2E), indicating a lack of DNA fragmentation of nuclei at the PMC stage. At the tetrad stage, the TUNEL-positive signal was marginally detectable in WT tapetal cells, but not in *7B-1*, suggesting the onset of PCD in WT tapetum (Fig 2B and 2F). At the binucleate microspore stage, strong TUNEL-positive signal was detected in WT tapetal cells (Fig 2C), while a lack of the signal in *7B-1* tapetum indicated a delay or failure of PCD in these cells (Fig 2G and 2H). At the mature pollen stage, TUNEL-positive signal was detected in WT anthers in fully degenerated tapetal cells (Fig 2D), while a weak signal observed in *7B-1* anthers in the vacuolated tapetal cells and collapsed microspores (Fig 2I). These observations demonstrated that PCD in WT tapetum has commenced at the tetrad stage, while in *7B-1* anthers the tapetum was failed to degenerate due to retardation or defect of PCD.

As mentioned earlier, free microspores could be marginally formed in very few of the *7B-1* anthers/lobes, while in most of them, they were arrested and lysed. Strong TUNEL-positive signal was detected in the arrested microspores, but not in the tapetal cells of either free or arrested microspores phenotypes (Fig 2G and 2H). To test if GA3 could restore the timely PCD in *7B-1* tapetal cells, *7B-1* buds were treated with GA3 at the panicle primordium stage. GA3 restored the PCD of tapetal cells in anthers/lobes, which produced free microspores, but not in those showing arrested microspores (Fig 2L and 2M). These observations confirmed that GA is essential for triggering of PCD in *7B-1* tapetal cells.

### Expression profiling revealed genes associated with male-sterile phenotype of *7B-1* anthers

**Fig 3:**
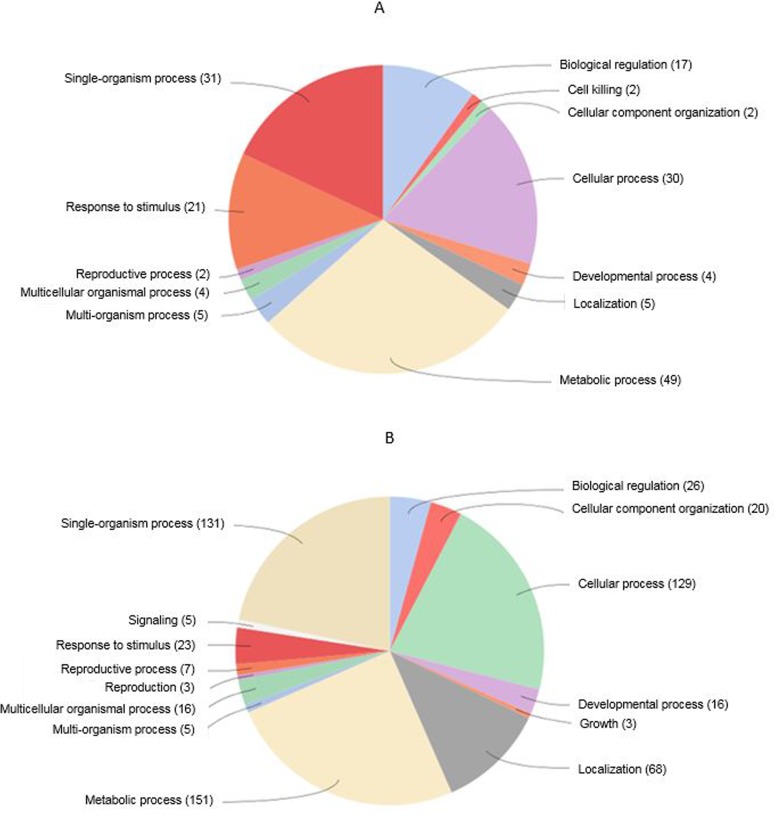
Gene ontology of DEGs. Up-regulated (A) and down-regulated (B) genes were categorized into different biological classes and numbers in the parenthesis indicate the frequency of members in each category.

Total RNA from anthers at three developmental stages of pre-meiosis, meiosis, and post-meiosis (designated as S1, S2, and S3) were pooled with equimolar ratio and used for construction of RNA-Seq libraries. Total of 14.1 and 13.9 million raw reads were sequenced for WT and *7B-1* libraries, respectively. After removal of short reads and rRNA matching reads, the clean reads were mapped (allowing 2 mismatches) to the tomato (cv. Heinz) reference genome ITAG v2.5. Read statistics are shown in Table 1. We identified 768 DEGs, including 132 up-regulated and 636 down-regulated genes (S1 Table). To gain insight into functional categories of DEGs, gene ontologies were assigned based on the biological processes using BLAST2GO (Fig 3). The majority of both up- and down-regulated genes corresponded to three major biological classes, including metabolic process, single-organism process and cellular process.

**Table 1:** Read statistics in WT and *7B-1* libraries.

Sample	Total	Adaptor trimming		Removal of rRNA-matching reads			Genome-matching reads	
		Clean	%Clean	rRNA-matching	Clean	%Clean	Mapped	%Mapped
WT	14,116,742	12,882,309	91.26	370,592	12,511,717	97.12	10,370,234	82.88
*7B-1*	13,927,913	12,696,565	91.16	351,302	12,345,263	97.23	10,319,965	83.59

**Fig 4:**
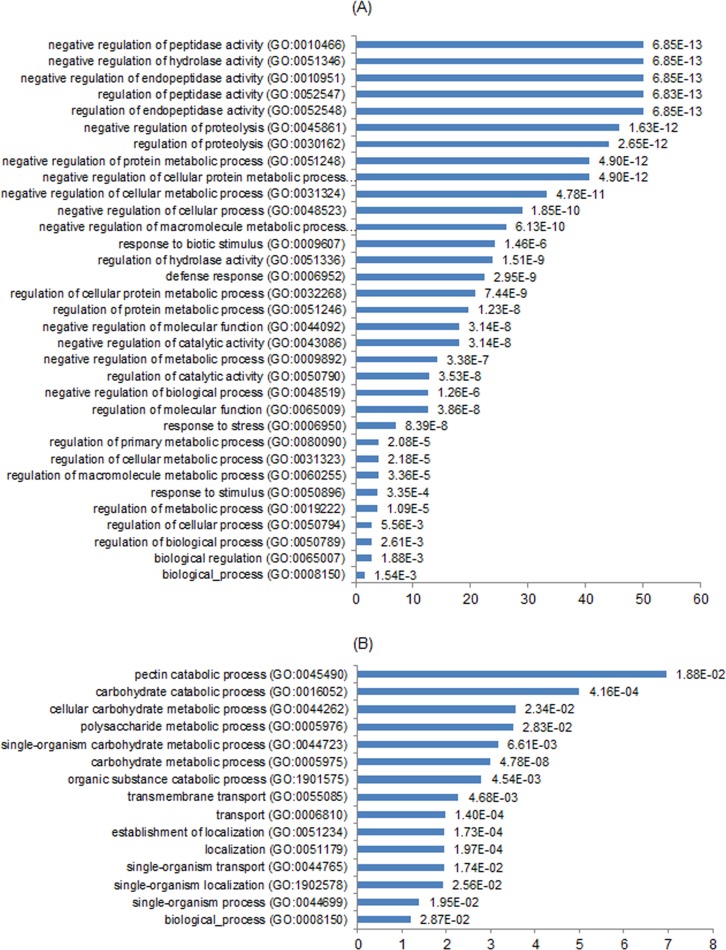
GO enrichment analysis of up- (A) and down-regulated (B) DEGs. Biological processes are listed on the Y-axis with their enrichment folds against all tomato genes (reference) presented on the X-axis. P-values are indicated for each GO term.

GO enrichment analysis was carried out in order to identify the major biological processes affected by the *7B-1* mutation. Thirty three and fifteen GO terms were over-represented (p<0.05) among up- and down-regulated DEGs, respectively (Fig 4). This indicates the broad effect of the *7B-1* mutation on transcriptional regulation of anther development, affecting diverse biological processes from regulation of proteolysis, defense response, response to stress to pectin catabolic and carbohydrate metabolic processes. Although several biological processes were enriched, nonetheless it was difficult to point a direct link between any of the enriched terms (with exception of the pectin catabolic process) and the male-sterile phenotype of *7B-1* anthers. Therefore, we focused our attention to DEGs with putative roles in regulation of anther development in *7B-1* mutant based on their expression, annotation and literature search. Sixteen candidates (Table 2) with key roles in regulation of meiosis, tapetum development, and cell-wall formation/degradation were further examined using qRT-PCR and *in situ* hybridization.

**Table 2:** List of DEGs with potential roles in anther development in *7B-1* mutant.

GeneID	Normalized reads		Statistics		Annotation
	WT	*7B-1*	DE	P-value	
Solyc10g079860.1.1	3.73	34.55	3.21	0.99	Beta-1,3-glucanase
Solyc04g005610.2.1	11.45	54.70	2.26	0.98	NAC transcription factor
Solyc00g071180.2.1	239.56	891.96	1.90	0.97	Cystatin
Solyc01g079200.2.1	32.79	152.49	1.70	0.98	Gibberellin 2-oxidase
Solyc05g052110.2.1	60.99	9.35	-2.74	0.99	Pectinesterase
Solyc06g008530.1.1	23.17	0.87	-4.64	0.99	Myosin XI
Solyc07g044870.2.1	358.24	13.27	-4.64	1.00	Polygalacturonase
Solyc12g098930.1.1	24.02	0.79	-5.06	0.99	Pyruvate dehydrogenase kinase
Solyc05g051250.2.1	271.66	8.71	-5.06	1.00	Glutamine synthetase
Solyc02g078370.1.1	307.04	9.23	-5.06	1.00	Anther-specific protein TA29
Solyc10g086460.1.1	291.50	9.83	-5.06	1.00	Actin
Solyc01g111540.2.1	172.06	5.29	-5.06	1.00	Beta-galactosidase
Solyc07g053460.2.1	75.02	1.37	-5.64	1.00	Cysteine proteinase
Solyc06g005180.1.1	33.48	0.63	-5.64	0.99	Zinc finger transcription factor
Solyc06g059970.2.1	204.97	2.67	-6.64	1.00	MADS-box transcription factor
Solyc06g059820.1.1	29.45	0.35	-6.64	0.99	F-box transcription factor

DE is differential expression values, which were calculated as log_2_-fold changes of the expression. Positive and negative values mean up- and down-regulation of expression in *7B-1*, respectively.

**Fig 5:**
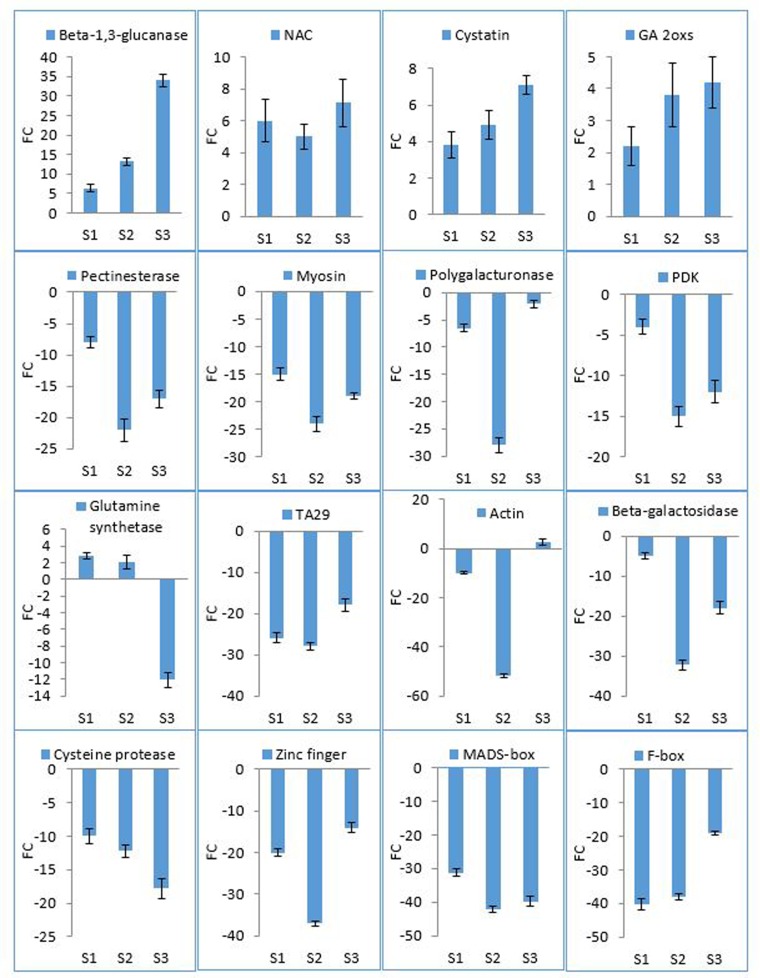
qRT-PCR analysis of DEGs in *7B-1* anthers. Expression changes are presented as normalized fold changes (FC) between *7B-1* and WT reference tissue. Positive and negative values indicate up- and down-regulation of the expression, respectively. Two-fold threshold was considered as a cutoff value for significant changes in the expression. Error bars represent standard errors of three biological replicates.

Candidate DEGs were validated using qRT-PCR at different developmental stages of *7B-1* anthers (Fig 5). Despite some quantitative differences in the expression levels, qRT-PCR results showed the same expression pattern as RNA-seq data. *Beta-1*,*3-glucanase* was up-regulated in S1, S2, and more strongly in S3. *NAC* was up-regulated in all stages. *Cystatin* and *gibberellin 2-oxidases* (*GA2ox*) were up-regulated with an increasing pattern during anther maturation. *Pectinesterase*, *myosin*, *polygalacturonase*, *pyruvate dehydrogenase kinase* (*PDK*), *beta-galactosidase*, and *zinc finger* were down-regulated in S1, S3, and more strongly in S2. *Glutamine synthetase* (*GS1*) was slightly up-regulated in S1 and S2, but strongly down-regulated in S3. *TA29* and *F-box* were down-regulated in S1 and S2, more strongly compared to S3. *Actin* was down-regulated in S1, very strongly in S2, but slightly up-regulated in S3. *Cysteine protease* was down-regulated S1, S2 and more strongly in S3. *MADS-box* was down-regulated more strongly in S2 and S3 compared to S1.

### Localization profile of DEGs in *7B-1* anthers

**Fig 6:**
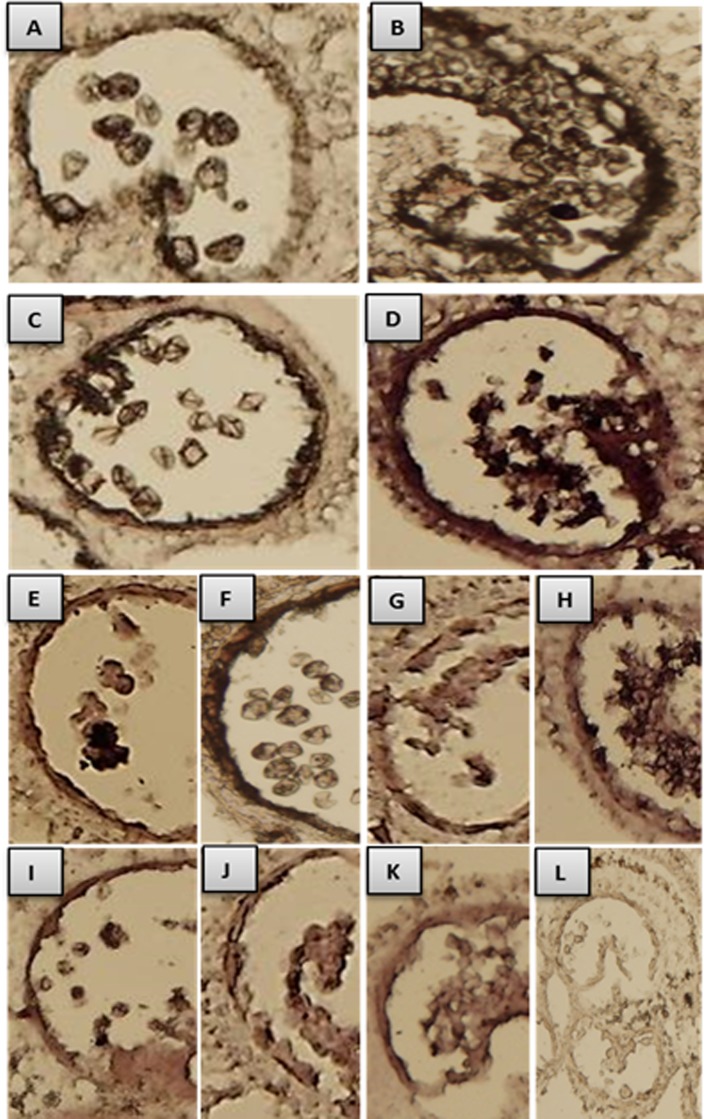
*In situ* localization of *beta-1*,*3-glucanase*, *GA2oxs*, *TA29* and *pectinesterase*. A and B: localization of *beta-1*, *3-glucanase* in WT and *7B-1* anthers respectively at binucleate microspores stage. C and D: *GA2ox* in WT and *7B-1* anthers at binucleate microspores stage, respectively. E and F: *TA29* in WT anthers at tetrads and binucleate microspores stages, respectively. G and H: *TA29* in *7B-1* anthers at tetrads and arrested binucleate microspores stages, respectively. I, J, K: *pectinesterase* in WT anthers at tetrads, in *7B-1* anthers at tetrads, and in *7B-1* anthers at arrested binucleate microspores stages, respectively. L: negative control, where a murine miR122a-specific probe was used to ensure that the experimental staining is not an artifact.

Fig 6 shows *in situ* localization of *beta-1*,*3 glucanase*, *GA2oxs*, *TA29*, and *pectinesterase* in WT and *7B-1* anthers. *Beta-1*,*3 glucanase* and *GA2oxs* were expressed in WT tapetum and binucleate microspores (Fig 6A and 6C), and more strongly in *7B-1* vacuolated tapetum and arrested microspores (Fig 6B and 6D). In WT anthers, *TA29* transcripts were localized in the tapetum, tetrads (Fig 6E), and the binucleate microspores (Fig 6F), while in *7B-1* anthers, they were localized in the tapetum, tetrads (Fig 6G), and the arrested microspores (Fig 6H). *Pectinesterase* transcripts were localized in the tapetum and the tetrads in both WT and *7B-1* anthers (Fig 6I and 6J) as well as in the arrested binucleate microspores in *7B-1* anthers (Fig 6K). The murine miR122a probe was used as negative control, which did not produce any hybridization signal (Fig 6L).

## Discussion

Despite the importance of male-sterility in hybrid seed breeding, the physiological mechanisms, i.e. nutritional, hormonal and environmental, which regulate the male-sterility are not yet fully understood. Until now, only a small number of genes have been identified that are specifically involved in this developmental process and the molecular mechanism of genetic male-sterility is still largely unknown. The transcriptomic profiling in our study showed differential expression of a large number of genes between WT and *7B-1* anthers. Majority of DEGs belonged to three major biological classes, including metabolic process, single-organism process and cellular process. This indicates that diverse gene regulation pathways are affected by or involved in the regulation of male-sterility in *7B-1* anthers. Further examination of GO terms showed enrichment of several biological processes, including those of special interest related to protein and carbohydrate metabolic processes. Several *pectinesterase* and *pectate lyase*-related genes were enriched within down-regulated DEGs, which were further characterized. Enrichment analysis suggested a broad impact of *7B-1* mutation primarily on the metabolism. Sixteen candidates were identified with potential roles in regulation of anther development and male-sterility in *7B-1* anthers and further characterized in different developmental stages between WT and *7B-1* anthers. These DEGs and their roles are discussed below.

During meiosis, tapetal cells undergo PCD and release beta-1,3-glucanase, which hydrolyses the callose from tetrads [51]. Persistent callose or delay in its dissolution could result in collapse of the developing microspores [52]. While callose was no longer detectable in the early microspore stage in WT anthers, it persisted around the tetrads and newly formed microspores in *7B-1* anthers, resulting in an arrested-microspore phenotype. A similar phenotype was observed in male-sterile anthers of *Brassica napus*, where callose was persistent around the tetrads [53]. qRT-PCR analysis showed up-regulation of *beta-1*,*3-glucanase* in *7B-1* anthers and *in situ* hybridization showed the prominent expression of this enzyme in *7B-1* tapetum at late stage of meiosis, where tapetal cells were vacuolated but not degenerated. Delay of tapetum degeneration in *7B-1* anthers could have led to *beta-1*,*3-glucanase* build-up level in these cells as detected by qRT-PCR and *in situ* hybridization signal, while callose around the newly formed microspores was not degraded, probably due to lack of the acting enzyme.

Several *pectinesterase* and *pectate lyase*-related genes were enriched within down-regulated DEGs. In addition to *pectinesterase*, several other cell wall modifying enzymes, including *beta-galactosidase*, a cellulose-modifying enzyme, and *polygalacturonase* which is a pectin-modifying enzyme [54,55] were strongly down-regulated in *7B-1* meiotic anthers. In *qrt1* and *qrt2* mutants of *Arabidopsis thaliana*, microspores were arrested as pectin was not degraded in primary cell walls around tetrads [56]. *Pectinesterase* transcripts were localized in tapetum, tetrads and arrested binucleate microspores in *7B-1* anthers. Suppression of the pectin-modifying enzymes in *7B-1* anthers were more pronounced during meiosis (stage S2), which could have impaired enzymatic degradation of cell wall pectin around tetrads, resulting in an arrested-microspores phenotype, similar to those observed in *qrt* mutants.

Previously, we found that *cystatin* and *cysteine protease* were up- and down-regulated in *7B-1* anthers, respectively with a pattern correlated to tapetum degeneration during anther development [41]. Similar results were observed using mRNA-seq and qRT-PCR in the present study. TUNEL assay showed a delay of PCD in *7B-1* tapetal cells. There results strongly suggest that suppression of *cysteine protease* could have caused a delay or defect of PCD in tapetal cells. GA plays an important role in floral organ growth, especially anther development. Tapetum is an important source of bioactive gibberellins in anthers [57], and alteration of GA level is often associated with abnormalities in anther development and male-sterility. GA-deficient mutants of tomato, rice and *Arabidopsis* exhibited common defects in PCD of tapetal cells, resulting in a post-meiotic arrest in male-sterile stamens [13,58,59]. In *sl-2* tomato mutant GA3 could restore the male-fertility [5,60]. Application of GA3 also partially restored the male-fertility in *7B-1* anthers (Omidvar et al., unpublished data). *GA2oxs* regulates the GA level through inactivation of endogenous bioactive GAs [61]. *7B-1* seedlings have a lower GA level compared to WT. Up-regulation of *GA2oxs* in *7B-1* anthers could have decreased the GA level in *7B-1* anthers, resulting in a defect in PCD of tapetal cells. Using TUNEL assay, we showed that application of GA3 restored the PCD of tapetal cells in *7B-1* anthers similar to those of WT, which suggests that GA3 is likely to regulate the initiation of PCD in tapetal cells.

Another gene which has been differentially expressed between WT and *7B-1* anthers was *TA29*. It is a tapetal-specific gene in tobacco, and its promoter region has been used for engineering of male-sterility in tobacco as well as other crops [62–65]. Although *TA29* is not functionally characterized with respect to regulation of male-sterility, silencing of this gene in tobacco has resulted in male-sterile transgenic plants, where tapetum was prematurely degenerated [65]. In our study *TA29* was strongly down-regulated in *7B-1* meiotic anthers, where the *TA29* transcripts were predominantly localized in the tapetal cells and tetrads and arrested binucleate microspores. Down-regulation of *TA29* in *7B-1* anthers did not result in premature degeneration of tapetum, but it could be associated with the defect of PCD in tapetal cells as it was strongly down-regulated and localized in undegenerated tapetal cells in late meiotic *7B-1* anthers.

Aberrant regulation of *actin*-, *tubulin*-, and *myosin*-related genes could disrupt the organization of actin and microtubules in meiotic cytoskeleton, thus leading to defective cytokinesis in developing pollens and male-sterility in crops [66,67]. In our study *actin* and *myosin* were down-regulated in *7B-1* anthers. In addition, *actin depolymerizing factors 3*/*10*, and *beta-tubulin* were also down-regulated in *7B-1* anthers (not validated by qRT-PCR). These observations indicate that the actin cytoskeleton balance may be disturbed in *7B-1* anthers, which could have directly affected the meiosis and pollen cell wall development. A case study showed that suppression of *pyruvate dehydrogenase kinase* in transgenic tobacco has led to tapetum perturbation and male-sterility [68]. The importance of *glutamine synthetase* in pollen reproduction has been shown in rice [69], maize [70], and tobacco [71]. Down-regulation of these two enzymes in *7B-1* anthers could also be associated with tapetum perturbation and meiosis break-down. In addition to the above mentioned genes, several transcription factors, including *F-box*, *MADS-box* and *zinc finger* genes were down-regulated, while *NAC* was up-regulated in *7B-1* anthers. Overexpression of *RMF* (*reduced male fertility*) gene, encoding a F-box protein in *Arabidopsis* caused the delay in tapetum degeneration and male-sterility [72]. Li et al. [73] showed that suppression of a F-box protein-encoding gene, *OsADF* (*anther development F-box*), perturbed tapetum degeneration and resulted in male-sterility in rice. *MADS-box* transcription factors play important roles in floral organ development, anther dehiscence and pollen maturation [74,75]. *Arabidopsis MS1* gene encodes PHD-type zinc finger protein, which is redundantly expressed in tapetum and regulates timely PCD in tapetal cells [11,76]. Several *NAC* transcription factors were differentially expressed between wild type and male-sterile flower buds of *Brassica rapa* [77]. *NACs* are key regulators of secondary wall thickening in anther tissue [78]. Although differential expression of these transcription factors in our study could be associated with the *7B-1* mutation and male-sterility phenotype, understating the exact function of these genes require further functional analysis.

A number of genes and transcription factors have been identified that control the tapetum formation and development [16,17,79–82]. However, little is known about the genetic basis regulating the PCD of tapetum during pollen development. In *Arabidopsis ms1* and rice *tdr* male-sterile mutants, tapetum aberrations were associated with failure or delay of PCD [32,76]. TUNEL assay in our study showed a delay of PCD in *7B-1* tapetal cells, where presence of large autophagic vacuolated tapetal cells at this stage suggested the necrotic-based breakdown of cells rather than the normal regulated PCD process. TUNEL-positive signal in arrested *7B-1* microspores was indicative of a PCD-based breakdown, likely as a result of the tapetum aberration. Treatment of GA-deficient male-sterile anthers of rice with GA3, restored the PCD of tapetal cells [13]. GA3 restored the PCD in *7B-1* anthers similar to those in WT, which suggest that GA3 is likely to regulate the PCD onset in *7B-1* anthers.

## Conclusions

**Fig 7:**
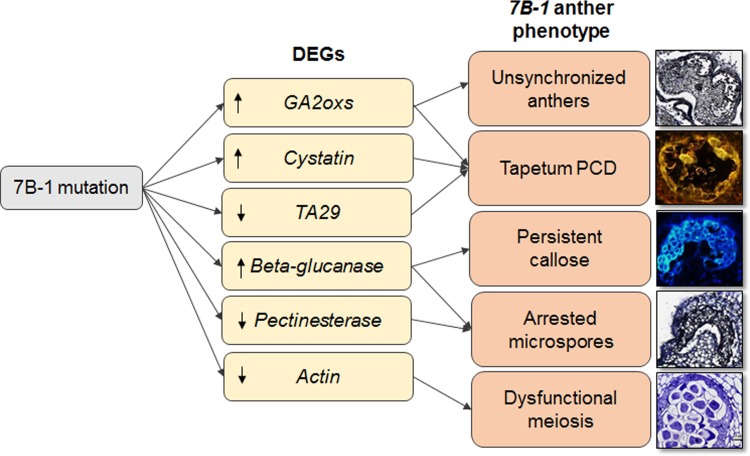
Schematic diagram of transcriptional regulation of male-sterility in *7B-1* anthers.

Overall in our study, we found that anther development and microsporogenesis in *7B-1* anthers was perturbed as evidenced by unsynchronized anther growth, dysfunctional meiosis, arrested microspores, defects in callose degradation, retarded PCD and abnormal tapetum profile. *In situ* localization signals for *beta-1*,*3 glucanase*, *GA2oxs*, *TA29*, and *pectinesterase* were coincided with qRT-PCR data, which confirmed the temporal gene expression results, suggesting that these genes could be closely related to tapetum development and regulation of meiosis in *7B-1* anthers. Our findings provide the first insights into the gene regulatory networks underlying the *7B-1* mutation and transcriptome dynamic between WT and *7B-1* anthers (Fig 7). It showed that *7B-1* mutation has predominantly affected genes regulating metabolic processes, and pointed out the distinct gene expression dynamic between *7B-1* and WT anthers. However, there is often a complex interplay of genes, transcription factors, hormonal balance, and environmental stimuli, which collaboratively regulate the male-sterility phenotypes and has to be taken into consideration.

## Supporting information

S1 Table List of differentially expressed genes. (DOCX)

S2 Table List of the primers used for qRT-PCR analysis. (DOCX)

S3 Table List of the DIG-labeled oligo-probes used for *in situ* hybridization. (XLSX)

## References

[1] Gorguet B, Schipper D, van Lammeren A, Visser RG, van Heusden AW. *ps-2*, the gene responsible for functional sterility in tomato, due to non-dehiscent anthers, is the result of a mutation in a novel polygalacturonase gene. Theor Appl Genet. 2009;118:1199–1209 10.1007/s00122-009-0974-9 19219598

[2] Emmanuel E, Levy AA. Tomato mutants as tools for functional genomics. Curr Opin Plant Biol. 2002;5:112–117 11856605 10.1016/s1369-5266(02)00237-6

[3] Lu Q, Li XH, Guo D, Xu CG, Zhang Q. Localization of *pms3*, a gene for photoperiod-sensitive genic male sterility, to a 284-kb DNA fragment. Mol Genet Genomics. 2005;273:507–511 10.1007/s00438-005-1155-4 15912317

[4] Gorman SW, McCormick S. Male sterility in tomato. Crit Rev Plant Sci. 1997;16:31–53

[5] Sawhney VK, Bhadula SK. Microsporogenesis in the normal and male-sterile stamenless-2 mutant of tomato. Can J Bot. 1988;66:2013–2021

[6] Rick CM. Genetics and development of nine male-sterile tomato mutants. Hilgardia. 1948;18:599–633

[7] Jeong HJ, Kang JH, Zhao M, Kwon JK, Choi HS, Hwan J, et al Tomato Male sterile 1035 is essential for pollen development and meiosis in anthers. J Exp Bot. 2014; 10.1093/jxb/eru389 PMC4246194 25262227

[8] Canales C, Bhatt AM, Scott R, Dickinson H. EXS, a putative LRR receptor kinase, regulates male germline cell number and tapetal identity and promotes seed development in *Arabidopsis*. Curr Biol. 2002;12:1718–1727 12401166 10.1016/s0960-9822(02)01151-x

[9] Zhao DZ, Wang GF, Speal B, Ma H. The *excess microsporocytes1* gene encodes a putative leucine-rich repeat receptor protein kinase that controls somatic and reproductive cell fates in the *Arabidopsis* anther. Genes Dev. 2002;16:2021–2031 10.1101/gad.997902 12154130 PMC186413

[10] Ito T, Nagata N, Yoshiba Y, Ohme-Takagi M, Ma H, Shinozaki K. Arabidopsis MALE STERILITY1 encodes a PHD-Type transcription factor and regulates pollen and tapetum development. Plant Cell. 2007;19:3549–3562 10.1105/tpc.107.054536 18032630 PMC2174881

[11] Yang C, Vizcay-Barrena G, Conner K, Wilson ZA. MALE STERILITY1 is required for tapetal development and pollen wall biosynthesis. Plant Cell. 2007;19:3530–3548 10.1105/tpc.107.054981 18032629 PMC2174882

[12] Aarts MG, Hodge R, Kalantidis K, Florack D, Wilson ZA, Mulligan BJ, et al The *Arabidopsis* male sterility 2 protein shares similarity with reductases in elongation/condensation complexes. Plant J. 1997;12:615–623 9351246 10.1046/j.1365-313x.1997.00615.x

[13] Aya K, Ueguchi-Tanaka M, Kondo M, Hamada K, Yano K, Nishimura M, Matsuoka M. Gibberellin modulates anther development in rice via the transcriptional regulation of GAMYB. Plant Cell. 2009;21:1453–1472 10.1105/tpc.108.062935 19454733 PMC2700530

[14] Millar AA, Gubler F. The Arabidopsis GAMYB-like genes, MYB33 and MYB65, are microRNA-regulated genes that redundantly facilitate anther development. Plant Cell. 2005;17:705–721 10.1105/tpc.104.027920 15722475 PMC1069693

[15] Zhang W, Sun Y, Timofejeva L, Chen C, Grossniklaus U, Ma H. Regulation of Arabidopsis tapetum development and function by DYSFUNCTIONAL TAPETUM1 (DYT1) encoding a putative bHLH transcription factor. Development. 2006;133: 3085–3095 10.1242/dev.02463 16831835

[16] Zhu J, Chen H, Li H, Gao JF, Jiang H, Wang C, et al *Defective in Tapetal development and function 1* is essential for anther development and tapetal function for microspore maturation in *Arabidopsis*. Plant J. 2008;55:266–277 10.1111/j.1365-313X.2008.03500.x 18397379

[17] Xu J, Yang C, Yuan Z, Zhang D, Gondwe MY, Ding Z, et al The ABORTED MICROSPORES regulatory network is required for postmeiotic male reproductive development in *Arabidopsis thaliana*. Plant Cell. 2010;22:91–107 10.1105/tpc.109.071803 20118226 PMC2828693

[18] Yang X, Wu D, Shi J, He Y, Pinot F, Grausem B, et al Rice CYP703A3, a cytochrome P450 hydroxylase, is essential for development of anther cuticle and pollen exine. J Integr Plant Biol. 2014; 10.1111/jipb.12212 24798002

[19] Wilson ZA, Morroll SM, Dawson J, Swarup R, Tighe PJ. The Arabidopsis MALE STERILITY1 (MS1) gene is a transcriptional regulator of male gametogenesis, with homology to the PHD-finger family of transcription factors. Plant J. 2001;28:27–39 11696184 10.1046/j.1365-313x.2001.01125.x

[20] Li H, Yuan Z, Vizcay-Barrena G, Yang C, Liang W, Zong, et al PERSISTENT TAPETAL CELL1 encodes a PHD-finger protein that is required for tapetal cell death and pollen development in rice. Plant Physiol. 2011;156(2):615–630 10.1104/pp.111.175760 21515697 PMC3177263

[21] Fu Z, Yu J, Cheng X, Zong X, Xu J, Chen M, et al The rice basic helix-loop-helix transcription factor TDR INTERACTING PROTEIN2 is a central switch in early anther development. Plant Cell 2014;26:1512–1524 10.1105/tpc.114.123745 24755456 PMC4036568

[22] Jung KH, Han MJ, Lee YS, Kim YW, Hwang I, Kim MJ, et al Rice Undeveloped Tapetum1 is a major regulator of early tapetum development. Plant Cell. 2005;17:2705–2722 10.1105/tpc.105.034090 16141453 PMC1242267

[23] Zhang DS, Liang WQ, Yuan Z, Li N, Shi J, Wang J, et al Tapetum degeneration retardation is critical for aliphatic metabolism and gene regulation during rice pollen development. Mol Plant. 2008;1:599–610 10.1093/mp/ssn028 19825565

[24] Niu N, Liang W, Yang X, Jin W, Wilson ZA, Hu J, et al EAT1 promotes tapetal cell death by regulating aspartic proteases during male reproductive development in rice. Nat Commun. 2013;4:1445 10.1038/ncomms2396 23385589

[25] Singh S, Sawhney VK. Cytokinins in a normal and the *ogura* (ogu) cytoplasmic male-sterile line of rapeseed (*Brassica napus*). Plant Sci. 1992;86:147–154

[26] Singh S, Sawhney VK, Pearce DW. Temperature effects on endogenous indole-3-acetic acid levels in leaves and stamens of the normal and male sterile ‘stamenless 2’ mutant of tomato. Plant Cell Environ. 1992;15:373–377

[27] Shukla A, Sawhney VK. Abscisic acid: one of the factors affecting male sterility in *Brassica napus*. Physiol Plantarum. 1994;91:522–528

[28] Smith MB, Horner HT, Palmer RG. Temperature and photoperiod effects on sterility in a cytoplasmicmale-sterile soybean. Crop Sci. 2001;41:702–704

[29] Guo RX, Sun DF, Tan ZB, Rong DF, Li CD. Two recessive genes controlling thermophotoperiod-sensitive male sterility in wheat. Theor Appl Genet. 2006;112:1271–1276 10.1007/s00122-006-0228-z 16465548

[30] Goldberg RB, Beals TP, Sanders PM. Anther development: basic principles and practical applications. Plant Cell. 1993;5:1217–1229 10.1105/tpc.5.10.1217 8281038 PMC160355

[31] Piffanelli P, Ross JHE, Murphy DJ. Biogenesis and function of the lipidic structures of pollen grains. Sex Plant Reprod. 1998;11:65–80

[32] Li N, Zhang DS, Liu HS, Yin CS, Li XX, Liang WQ, et al The rice tapetum degeneration retardation gene is required for tapetum degradation and anther development. Plant Cell. 2006;18: 2999–3014 10.1105/tpc.106.044107 17138695 PMC1693939

[33] Sawhney VK. Genic male sterility In, Shivanna KR Sawhney VK editors Pollen biotechnology for crop production and improvement. Cambridge, Cambridge University Press pp. 1997;183–198

[34] Sawhney VK. Photoperiod-sensitive male-sterile mutant in tomato and its potential use in hybrid seed production. J Hortic Sci Biotech. 2004;79:138–141

[35] Fellner M, Zhang R, Pharis RP, Sawhney VK. Reduced de-etiolation of hypocotyl growth in a tomato mutant is associated with hypersensitivity to and high endogenous levels of abscisic acid. J Exp Bot. 2001;52:725–738 11413209 10.1093/jexbot/52.357.725

[36] Fellner M, Sawhney VK. The 7B-1 mutant in tomato shows blue-light-specific resistance to osmotic stress and abscisic acid. Planta. 2002;214:675–682 10.1007/s004250100671 11882935

[37] Bergougnoux V, Zalabak D, Jandova M, Novak O, Wiese-Klinkenberg A, Fellner M. Effect of blue light on endogenous isopentenyladenine and endoreduplication during photomorphogenesis and de-etiolation of tomato *Solanum lycopersicum* L seedlings. PLoS One. 2012;7:e45255 10.1371/journal.pone.0045255 23049779 PMC3458014

[38] Sheoran IS, Rossb A, Olsonb D, Sawhney VK. Differential expression of proteins in the wild type and 7B-1 male-sterile mutant anthers of tomato *Solanum lycopersicum*), A proteomic analysis. J Proteomics. 2009;71:624–636 10.1016/j.jprot.2008.10.006 19032992

[39] Omidvar V, Fellner M. DNA methylation and transcriptomic changes in response to different lights and stresses in *7B-1* male-sterile tomato. PLoS ONE. 2015;10: e0121864 10.1371/journal.pone.0121864 25849771 PMC4388563

[40] Omidvar V, Mohorianu I, Dalmay T, Fellner M. Identification of miRNAs with potential roles in regulation of anther development and male-sterility in 7B-1 male-sterile tomato mutant. BMC Genomics. 2015a;16:878 10.1186/s12864-015-2077-0 26511108 PMC4625851

[41] Omidvar V, Mohorianu I, Dalmay T, Fellner M. MicroRNA regulation of abiotic stress response in 7B-1 male-sterile tomato mutant. Plant Genome. 2015b; 10.3835/plantgenome2015.02.0008 33228265

[42] Bolger AM, Lohse M, Usadel B. Trimmomatic: a flexible trimmer for Illumina sequence data. Bioinformatics. 2014;30:2114–2120 10.1093/bioinformatics/btu170 24695404 PMC4103590

[43] Quast C, Pruesse E, Yilmaz P, Gerken J, Schweer T, Yarza P, et al The SILVA ribosomal RNA gene database project: improved data processing and web-based tools. Nucleic Acids Res. 2013;41: D590–6 10.1093/nar/gks1219 23193283 PMC3531112

[44] Langmead B, Trapnell C, Pop M, Salzberg SL. Ultrafast and memory-efficient alignment of short DNA sequences to the human genome. Genome Biol. 2009;10: R25 10.1186/gb-2009-10-3-r25 19261174 PMC2690996

[45] Kim D, Pertea G, Trapnell C, Pimentel H, Kelley R, Salzberg SL. TopHat2: accurate alignment of transcriptomes in the presence of insertions, deletions and gene fusions. Genome Biol. 2013;14: R36 10.1186/gb-2013-14-4-r36 23618408 PMC4053844

[46] Robinson MD, Oshlack A. A scaling normalization method for differential expression analysis of RNA-seq data. Genome Biol. 2010;11:R25 10.1186/gb-2010-11-3-r25 20196867 PMC2864565

[47] Tarazona S, García-Alcalde F, Dopazo J, Ferrer A, Conesa A. Differential expression in RNA-seq: a matter of depth. Genome Res. 2011;21: 2213–2223 10.1101/gr.124321.111 21903743 PMC3227109

[48] Mohorianu I, Schwach F, Jing R, Lopez-Gomollon S, Moxon S, Szittya G, et al Profiling of short RNAs during fleshy fruit development reveals stage-specific sRNAome expression patterns. Plant J. 2011;67:232–246 10.1111/j.1365-313X.2011.04586.x 21443685

[49] Mi H, Muruganujan A, Casagrande JT, Thomas PD. Large-scale gene function analysis with the PANTHER classification system. Nat Protocol. 2013;8:1551–1566 10.1038/nprot.2013.092 PMC6519453 23868073

[50] Livak KJ, Schmittgen TD. Analysis of relative gene expression data using real-time quantitative PCR and the 2(-Delta Delta C(T)). Methods. 2001;25:402–408 10.1006/meth.2001.1262 11846609

[51] Scott RJ, Spielman M, Dickinson HG. Stamen structure and function. Plant Cell. 2004;16(Suppl): S46–S60 15131249 10.1105/tpc.017012 PMC2643399

[52] Lu P, Maofeng C, Jiange Y, Gang N, Guoliang W, Hong M. The Arabidopsis CALLOSE DEFECTIVE MICROSPORE1 gene is required for male fertility through regulating callose metabolism during microsporogenesis. Plant Physiol. 2014;164:1893–1904 10.1104/pp.113.233387 24567187 PMC3982751

[53] Zhu Y, Xiaoling D, Zhengfu Z, Shengqian X, Bin Y, Wen J, et al Separation defect of tapetum cells and microspore mother cells results in male sterility in Brassica napus: the role of abscisic acid in early anther development. Plant Mol Biol. 2010;72:111–123 10.1007/s11103-009-9556-0 19862484

[54] Nakamura A, Maeda H, Mizuno M, Koshi Y, Nagamatsu Y. beta-Galactosidase and its significance in ripening of "Saijyo" Japanese Persimmon fruit. Biosci Biotechnol Biochem. 2003;67:68–76 10.1271/bbb.67.68 12619675

[55] Lazan H, Ng SY, Goh LY, Ali ZM. Papaya beta-galactosidase/galactanase isoforms in differential cell wall hydrolysis and fruit softening during ripening. Plant Physiol Biochem. 2004;42(11):847–53 10.1016/j.plaphy.2004.10.007 15694277

[56] Rhee SY, Somerville CR. Tetrad pollen formation in quartet mutants of *Arabidopsis thaliana* is associated with persistence of pectic polysaccharides of the pollen mother cell wall. Plant J. 1998; 15:79–88 9744097 10.1046/j.1365-313x.1998.00183.x

[57] Kaneko M, Itoh H, Inukai Y, Sakamoto T, Ueguchi-Tanaka M, Ashikari M, et al Where do gibberellin biosynthesis and gibberellin signaling occur in rice plants?. Plant J. 2003;35:104–115 12834406 10.1046/j.1365-313x.2003.01780.x

[58] Jacobsen SE, Olszewski NE. Characterization of the arrest in anther development associated with gibberellin deficiency of the *gib-1* mutant of tomato. Plant Physiol. 1991;97:409–414 16668400 10.1104/pp.97.1.409 PMC1081013

[59] Plackett ARG, Powers SJ, Fernandez-Garcia N, Urbanova T, Takebayashi Y, Seo M, et al Analysis of the developmental roles of the Arabidopsis gibberellin 20-oxidases demonstrates that GA20ox1, -2, and -3 are the dominant paralogs. Plant Cell. 2012;24:941–960 10.1105/tpc.111.095109 22427334 PMC3336139

[60] Rastogi R, Sawhney VK. Flower Culture of a Male Sterile Stamenless-2 Mutant of Tomato (Lycopersicon esculentum). Amer J Bot. 1988; 75:513–518.

[61] Ross JJ, Reid JB, Swain SM, Hasan O, Poole AT, Hedden P, et al Genetic regulation of gibberellin deactivation in *Pisum*. Plant J. 1995;7: 513–523

[62] Koltunow AM, Truettner J, Cox KH, Wallroth M, Goldberg RB. Different temporal and spatial gene expression patterns occur during anther development. Plant Cell. 1990;2:1201–1224 10.1105/tpc.2.12.1201 12354953 PMC159967

[63] Mariani C, De Beuckeleer M, Truettner J, Leemans J, Goldberg RB. lnduction of male sterility in plants by a chimaeric ribonuclease gene. Nature. 1990;347:737–741

[64] Rong Z, Yu-le L, Feng Z, Sheng-guo L, Liang-yi K, Peng L. Induction of male sterility in oilseed rape by TA29-barnase gene. Acta Botanica Sinica. 1996;38:582–585

[65] Nawaz-ul-Rehman MS, Mansoor S, Khan AA, Zafar Y, Briddon RW. RNAi-mediated male sterility of tobacco by silencing TA29. Mol Biotechnol. 2007;36(2):159–165 17914195 10.1007/s12033-007-0025-1

[66] Zhang J, Zhang C, Cheng Y, Qi L, Wang S, Hou X. Microtubule and male sterility in a gene-cytoplasmic male sterile line of non-heading Chinese cabbage. J Sci Food Agric. 2012;92:3046–3054 10.1002/jsfa.5722 22581783

[67] Xu C, Liu Z, Zhang L, Zhao C, Yuan S, Zhang F. Organization of actin cytoskeleton during meiosis I in a wheat thermo-sensitive genic male sterile line Protoplasma. 2013;250:415–422 10.1007/s00709-012-0386-6 22350736

[68] Yui R, Iketani S, Mikami T, Kubo T. Antisense inhibition of mitochondrial pyruvate dehydrogenase subunit in anther tapetum causes male sterility. Plant J. 2003;34:57–66 12662309 10.1046/j.1365-313x.2003.01704.x

[69] Tabuchi M, Sugiyama K, Ishiyama K, Inoue E, Sato T, Takahashi H, et al Severe reduction in growth rate and grain filling of rice mutants lacking OsGS1;1, a cytosolic glutamine synthetase1;1. Plant J. 2005;42: 641–651 10.1111/j.1365-313X.2005.02406.x 15918879

[70] Martin A, Lee J, Kichey T, Gerentes D, Zivy M, Tatout C, et al Two cytosolic glutamine synthetase isoforms of maize are specifically involved in the control of grain production. Plant Cell. 2006;18:3252–3274 10.1105/tpc.106.042689 17138698 PMC1693956

[71] Mamun AN. Reversible male sterility in transgenic tobacco carrying a dominant-negative mutated glutamine synthetase gene under the control of microspore-specific promoter. Indian J Exp Biol. 2007;45:1022–1030 18254207

[72] Kim OK, Jung JH, Park CM. An Arabidopsis F-box protein regulates tapetum degeneration and pollen maturation during anther development. Planta. 2010;232:353–366 10.1007/s00425-010-1178-x 20458496

[73] Li L, Li Y, Song S, Deng H, Li N, Fu X, et al An anther development F-box (ADF) protein regulated by tapetum degeneration retardation (TDR) controls rice anther development. Planta. 2015;241:157–166 10.1007/s00425-014-2160-9 25236969

[74] Schreiber DN, Bantin J, Dresselhaus T. The MADS box transcription factor ZmMADS2 is required for anther and pollen maturation in maize and accumulates in apoptotic bodies during anther dehiscence. Plant Physiol. 2004;134:1069–1079 10.1104/pp.103.030577 15001699 PMC389931

[75] Huang F, Xu G, Chi Y, Liu H, Xue Q, Zhao T, et al A soybean MADS-box protein modulates floral organ numbers, petal identity and sterility. BMC Plant Biol. 2014;14:89 10.1186/1471-2229-14-89 24693922 PMC4021551

[76] Vizcay-Barrena G, Wilson ZA. Altered tapetal PCD and pollen wall development in the Arabidopsis *ms1* mutant. J Exp Bot. 2006;57:2709–2717 10.1093/jxb/erl032 16908508

[77] Dong X, Feng H, Xu M, Lee J, Kim YK, Lim YP, et al Comprehensive analysis of genic male sterility-related genes in *Brassica rapa* using a newly developed Br300K Oligomeric Chip. PLoS ONE. 2013;8(9): e72178 10.1371/journal.pone.0072178 24039743 PMC3770635

[78] Distelfeld A, Pearce SP, Avni R, Scherer B, Uauy C, Piston F, et al Divergent functions of orthologous NAC transcription factors in wheat and rice. Plant Mol Biol. 2012;78:515–524 10.1007/s11103-012-9881-6 22278768 PMC4773031

[79] Sun YJ, Hord CLH, Chen CB, Ma H. Regulation of *Arabidopsis* early anther development by putative cell-cell signaling molecules and transcriptional regulators J Integr Plant Biol. 2007;49:60–68

[80] Liu Z, Bao E, Liang W, Yin J, Zhang D. Identification of gamyb-4 and analysis of the regulatory role of GAMYB in rice anther development. J Integr Plant Biol. 2010;52:670–678 10.1111/j.1744-7909.2010.00959.x 20590996

[81] Zhu J, Lou Y, Xu X, Yang ZN. A genetic pathway for tapetum development and function in *Arabidopsis*. J Integr Plant Biol. 2011;53:892–900 10.1111/j.1744-7909.2011.01078.x 21957980

[82] Gu JN, Zhu J, Yu Y, Teng XD, Lou Y, Xu XF. DYT1 directly regulates the expression of TDF1 for tapetum development and pollen wall formation in Arabidopsis. Plant J. 2014;80:1005–1013 10.1111/tpj.12694 25284309

