# Supplementary material for: Transcriptional regulation of male-sterility in 7B-1 male-sterile tomato mutant
Source: PLoS One. 2017 Feb 8;12(2):e0170715. doi: 10.1371/journal.pone.0170715 (PMC5298235; doi:10.1371/journal.pone.0170715)
Supplement: S1 Table — (DOCX) [file pone.0170715.s001.docx]

**S1 Table**. List of the primers used for qRT-PCR analysis.

| **Target candidate** | **Accession no.** | **Forward Primer (5'-3')** | **Reverse primer (5'-3')** |
| --- | --- | --- | --- |
| *Beta-1,3-glucanase* | Solyc10g079860.1.1 | AGACAACGTCCGAGGGTATG | TTTTTCAAGGGCCGAGTATG |
| *NAC transcription factor* | Solyc04g005610.2.1 | GGGCAGCTGTGTCTGGTTAT | CTCCATGGCTTGATGAGGCA |
| *Cystatin* | Solyc00g071180.2.1 | GCAGTGATGGCAAACGTAGG | CACCTTCAGTGCCCTCAAAT |
| *Gibberellin 2-oxidase* | Solyc01g079200.2.1 | GGCTCCCTTTGGTTATGGTAG | GTCACCGAGCTGAAAGTAGAG |
| *Pectinesterase* | Solyc05g052110.2.1 | GGCAATGGTGACAGCACAAG | AGCGTTTTAAAGTAGCACCACAT |
| *Myosin XI* | Solyc06g008530.1.1 | GTCTTGGAAGGGAATGCCGA | GCCAAGTGTCCACGCAATTT |
| *Polygalacturonase* | Solyc07g044870.2.1 | CAGGTCAGCCAGGAGTGTTT | ACTCAAGTGGTGCCTTGCAT |
| *Pyruvate dehydrogenase kinase* | Solyc12g098930.1.1 | AGGCACAATAACGTCGTTCC | ACAGTTGGGAAGTGGATTCG |
| *Glutamine synthetase* | Solyc05g051250.2.1 | TTGGTCCTGCTGTTGGTATC | CCAATCACCAGGGATAGGTTT |
| *Anther-specific protein TA29* | Solyc02g078370.1.1 | GGTCATCAGTCAGAGCAGCA | GGTCGGGGATATTGTTTGTG |
| *Actin* | Solyc10g086460.1.1 | CACTTCCTCACGCTATCCTTC | TTCCTTCTCAGCACTGGTTG |
| *Beta-galactosidase* | Solyc01g111540.2.1 | ATGGAACATCCGCTACAGAAG | GAGTTCTTTGAAACCAGAAGC |
| *Cysteine proteinase* | Solyc07g053460.2.1 | TGCGGACATGACAAATCGAGA | ACAACCCCATCCTTCCTCCA |
| *Zinc finger transcription factor* | Solyc06g005180.1.1 | GCAAAATGAGTGAAAATGGTGGAT | GTGACTCGCTCTATGTCCCC |
| *MADS-box transcription factor* | Solyc06g059970.2.1 | TTCTAGTATCAGTGCCAAGCAG | CCTCTCATATTTCCACTTCCACCA |
| *F-box transcription factor* | Solyc06g059820.1.1 | GGTGGAACCCTGCTACTAAAG | CATCATACCCTACCGCTGTTAC |
| *α-tubulin* | Solyc04g077020.2.1 | TGAGGTCTTCTCACGCATTGACCA | AATCCTTCTCGAGGGCAGCAAGAT |
| *CAC* | SGN-U314153 | CCTCCGTTGTGATGTAACTGG | ATTGGTGGAAAGTAACATCATCG |
