# Supplementary material for: Transcriptional regulation of male-sterility in 7B-1 male-sterile tomato mutant
Source: PLoS One. 2017 Feb 8;12(2):e0170715. doi: 10.1371/journal.pone.0170715 (PMC5298235; doi:10.1371/journal.pone.0170715)
Supplement: S2 Table — (DOCX) [file pone.0170715.s002.docx]

**S2 Table**. List of the DIG-labeled oligo-probes used for *in situ* hybridization.

| **Target candidate** | **Probe sequence (5'-3')** |
| --- | --- |
| *Beta-1,3-glucanase* | TTTTTCAAGGGCCGAGTATG |
| *GA2oxs* | TCAATCTCTGAGCATGGCGG |
| *TA29* | TGTCGTGCTGGTCTAACTGAT |
| *Pectinesterase* | AGCGTTTTAAAGTAGCACCACAT |
| miR122a | TGGAGTGTGACAATGGTGTTTG |
